# Structural Characteristics and Properties of Redissolved Silk Sericin

**DOI:** 10.3390/polym15163405

**Published:** 2023-08-14

**Authors:** Hye Gyeoung Lee, Mi Jin Jang, Byung-Dae Park, In Chul Um

**Affiliations:** 1 Department of Biofibers and Biomaterials Science, Kyungpook National University, Daegu 41566, Republic of Korea; 2 Preclinical Research Center, Daegu-Gyeongbuk Medical Innovation Foundation, Daegu 41061, Republic of Korea; 3 Department of Wood and Paper Science, Kyungpook National University, Daegu 41566, Republic of Korea

**Keywords:** sericin, dissolution behavior, structural characteristics, molecular weight

## Abstract

Silk sericin has garnered the attention of researchers as a promising biomaterial because of its good biocompatibility and high water retention. However, despite its useful properties, the poor storage stability of sericin has restricted its extensive use in biorelated applications. This study extracted sericin from silkworm cocoon, dried and stored it as a solid, and then dissolved it in hot water conditions to improve the storage stability of sericin for its use. The dissolution behavior of the extracted sericin solids was examined in conjunction with the structural characteristics and properties of dissolved sericin. Consequently, the results of solution viscosity, gel strength, crystallinity index, and thermal decomposition temperature indicated that the molecular weight (MW) of the dissolved sericin remained constant until a dissolution time of 5 min, following which deterioration was observed. The optimum condition of dissolution of the extracted sericin solid was 5 min at 90 °C. Conclusively, the extracted sericin could be stored in a dry state and dissolved to prepare redissolved sericin aqueous solution with the same MW as extracted sericin, thereby improving the storage stability of the sericin aqueous solution.

## 1. Introduction

Silk is composed of two biopolymers: fibroin and sericin. Sericin covers two strands of fibroin and is removed during the degumming process to enhance the touch feeling and luster of silk textiles [1,2]. The recently reported useful properties of sericin have garnered the attention of researchers involved in biomedical and cosmetic fields. Sericin exhibits a good wound-healing effect [3], good water retention [4], antioxidant effects [5,6], antibacterial properties [7,8,9], and functions as a good ultraviolet light block [10]. Consequently, several studies have focused on sericin for various cosmetic and biomedical applications, including wound dressing [11], tissue engineering scaffolds [12], and mask packs [13,14,15,16].

However, sericin aqueous solutions can easily turn into a gel and thus rot. This indicates the low storage stability of sericin solutions. Consequently, 50,000 tons of silk sericin are abandoned annually worldwide [1]. In addition, when silk sericin is extracted from raw silk fiber and dried, the sericin solid readily crystallizes [17], rendering its dissolution in water challenging. Thus, the use of sericin solid via dissolution is difficult. Moreover, sericin experiences hydrolytic molecular decomposition in hot water, which decreases the molecular weight (MW) [18,19,20,21]. This leads to the deterioration of the mechanical properties of silk sericin film and gel [20,21,22,23], further impacting its applicability to cosmetic and biomedical applications.

Despite the difficulties regarding the dissolution of extracted sericin solids, this method can effectively solve the problem of low storage of sericin aqueous solutions. Specifically, the sericin solution does not become a gel and rot when it is dried to a solid state, thereby prolonging its storage period. Moreover, it can be used via dissolution when required. However, despite the utilization of dissolution of sericin, a related study has not yet been conducted.

This study extracted sericin from a silkworm cocoon (i.e., raw silk) and dried it to prepare “extracted sericin solid”. Subsequently, it was dissolved to prepare a sericin aqueous solution (redissolved sericin solution). We observed the dissolution behavior of the extracted sericin solid and examined the effect of the dissolution time on the MW, structure, and properties of the redissolved silk sericin solid.

## 2. Experimental Section

### 2.1. Extraction and Redissolution of Silk Sericin

*Bombyx mori* Baekokjam silkworm cocoons were obtained from the National Institute of Agricultural Science (Wanju, Republic of Korea) and used to extract (i.e., dissolve) silk sericin. The silkworm cocoons were immersed in purified water and treated at 120 °C for 30 min using an autoclave (JSAC-60, JSR, Gongju, Republic of Korea). Consequently, raw sericin in silkworm cocoons dissolved in the hot water, resulting in the sericin aqueous solution. The purified water was produced using a water purification system (RO50, Hana Science, Seongnam, Republic of Korea). The ratio of cocoon and purified water was set at 1:25 (*w*/*v*). Following the extraction (first dissolution), the sericin aqueous solution was filtered using a polyester nonwoven fabric to prepare a 1% (*w*/*w*) aqueous sericin solution [24]. Subsequently, it was poured into Petri dishes and dried at 60 °C in a drying oven (WOF-50, Daihan Scientific, Wonju, Republic of Korea) to prepare an extracted sericin film. The sericin powder and film, prepared via first dissolution (i.e., extraction), are referred to as “extracted” sericin powder and film, respectively. The extracted sericin film was ground to produce the extracted sericin powder, which was then dissolved in purified water at 90 °C for different time intervals (30 s, 1 min, 5 min, 10 min, and 30 min) to prepare a sericin aqueous solution. Thereafter, the solution was filtered by a filter paper (WF1-0900, Whatman, Maidstone, United Kingdom) with a pore size of 11 μm and poured into Petri dishes and dried again at 60 °C in the drying oven to prepare the sericin films. The sericin powder and film, prepared by dissolving the extracted sericin, are referred to as “redissolved” sericin powder and film, respectively. We used the expressions of “redissolution” or “redissolved” in this study because raw sericin in a cocoon was first dissolved (i.e., extracted) in hot water, following which the extracted sericin was dissolved in hot water to prepare another sericin (i.e., redissolved sericin).

### 2.2. Measurement and Characterization

To determine the solubility of the extracted sericin powder, it was redissolved in hot water at different temperatures (85 °C, 90 °C, 95 °C, and 100 °C) for different time intervals (30 s, 1 min, 2 min, 3 min, 5 min, 10 min, and 30 min) and subsequently filtered using a filter paper (WF1-0900, Whatman, Maidstone, United Kingdom) with a pore size of 11 μm. The solubility of the extracted sericin powder was calculated, as shown in Equation (1). The dry weight of the sericin powders was measured using a moisture analyzer (XM60, Precisa Gravimetrics AG, Dietikon, Switzerland).

(1) $$Solubility\;\left(\%\right)=\frac{W_{1}-W_{2}}{W_{1}}\times 100$$

where:

W_1_: dry weight of extracted sericin powder (i.e., sericin powder before redissolution);
W_2_: dry weight of redissolved sericin powder (i.e., sericin powder after redissolution).

The shear viscosity was examined by performing the steady-state flow test using a rheometer (MARS III, Thermo Fisher Scientific, Karlsruhe, Germany) at 25 °C. A cone and 60 mm plate geometry was used. The radius and angle of the cone were 60 mm and 1°, respectively. The tested shear rate range was 0.55–100 s^−1^. Furthermore, to measure the shear viscosity, the sericin powders were dissolved in 98% formic acid at 55 °C for 30 min to prepare a 0.3% (*w*/*w*) sericin/formic acid solution. The gel strength was measured via an axial test using the rheometer 35 mm plate geometry at 25 °C. The 35 mm plate of the rheometer was capable of compressing a sample in the range of 13–1 mm in height from the 35 mm plate at a speed of 0.2 mm/s [14,21]. To measure the gel strength, the sericin was dissolved in 98% formic acid at 55 °C for 45 min to prepare 1.5% (*w*/*w*) sericin/formic acid solutions, which were stored at 4 °C for 3 days to fabricate the sericin gels [25].

The molecular conformations of sericin were investigated using Fourier transform infrared spectroscopy (FTIR; Nicolet 380, Thermo Fisher Scientific, Waltham, MA, USA) with the attenuated total reflection (ATR). The scan range was 4000–650 cm^−1^. The scan number and resolution were 32, and 8 cm^−1^, respectively. Furthermore, the crystallinity index was obtained by considering the absorbance ratio of the peaks at 1616 and 1643 cm^−1^ in the FTIR spectrum using Equation (2) [26]. The FTIR measurements were conducted seven times to obtain the average and standard deviations of the crystallinity index.

(2) $$Crystallinity\;index\;\left(\%\right)=\frac{A_{1616{cm}^{-1}}}{A_{1616{cm}^{-1}}+A_{1643{cm}^{-1}}}\times 100,$$

where:

A_1616cm_^−1^: the absorbance at 1616 cm^−1^ attributed to the β-sheet conformation in the crystalline region;
A_1643cm_^−1^: the absorbance at 1643 cm^−1^ corresponds to the random coil in the amorphous region.

To evaluate the water absorption ability of the silk sericin powders, they were maintained at standard conditions (20 °C and 65% relative humidity) for more than 24 h, and their moisture regain values were calculated using Equation (3) [27,28]. The dry weight of the silk sericin powder was determined using the moisture analyzer.

(3) $$Moisture\;regain\left(\%\right)=\frac{Initial\;weight-Dry\;weight}{Dry\;weight}\times 100$$

Differential scanning calorimetry (DSC) analysis was conducted using the Thermal Analysis Instrument Q 10 (DS25, TA Instrument, New Castle, DE, USA). The tested temperature range was 60–265 °C, and the scanning rate was controlled to 10 °C/min. The analysis was performed using a 50 mL/min nitrogen gas flow [29].

The in vitro cytotoxicity test of the silk sericin was conducted using an extraction method in accordance with ISO 10993-5. Before the extraction, the silk sericins were sterilized with an ethylene oxide gas. Subsequently, the extraction was conducted by immersing the silk sericin (6 × 3 cm^2^) in a 6 mL RPMI1640 culture medium.

The cytotoxicity of the silk sericin on the L929 cells was determined via the application of the CCK-8 (Cell Counting Kit 8, Dojindo, Japan) assay in vitro. Following 24 and 48 h of incubation, the absorbance was measured at 450 nm. Finally, the cell viability of the silk sericin was calculated using Equation (4) [30].

(4) $$Cell\;viability\;\left(\%\right)=\frac{{OD}_{exp}-{OD}_{blank}}{{OD}_{control}-{OD}_{blank}}\times 100$$

The cytotoxicity of silk sericin was also evaluated by conducting fluorescence staining using a live/dead viability/cytotoxicity kit (L3224, Invitrogen, Waltham, MA, USA), as per the manufacturer’s protocol. After 24 and 48 h of incubation, the extracts were discarded, and 300 µL of a staining solution was added to each well. After 45 min of incubation, the staining solutions were eliminated, and the cells were observed under a fluorescence-inverted microscope (IX83, Olympus, Tokyo, Japan).

## 3. Results and Discussion

### 3.1. Solubility of Extracted Sericin

**Figure 1:**
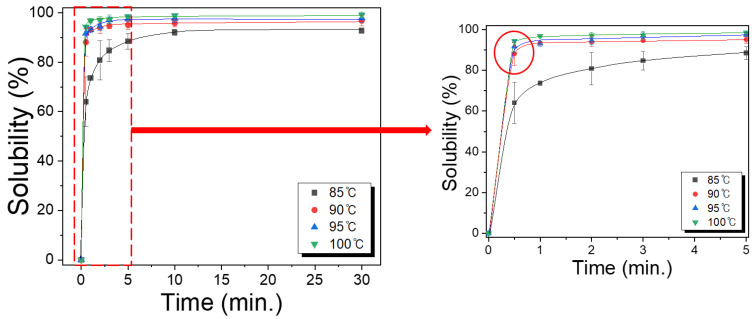
Effect of time and temperature on the solubility of extracted sericin solid (n = 3).

Figure 1 shows the solubility of the extracted sericin solid in water at different temperatures at various times. As is evident, the solubility of the extracted sericin increased remarkably until 1 min and then plateaus. Further, the solubility increased with an increase in the dissolution temperature. At a dissolution temperature of 85 °C, the solubility reached 73.6% at a dissolution time of 1 min. Thereafter, it reached 88.4% and 92.7% at 5 and 30 min, respectively. However, at a temperature of 90 °C, the solubility rose to 93.3% at 1 min, reaching 95.1% at 5 min. A further increase in the dissolution temperature exhibited a minimal change in the solubility, although it increased slightly with the dissolution temperature. This result, indicating that more than 90% of the extracted sericin could be dissolved at 90 °C and for a time greater than 1 min, is highly encouraging. However, the occurrence of hydrolytic molecular decomposition of silk sericin in hot water decreased the MW and the deterioration of the mechanical properties of sericin [20,21]. This implies that the increase in dissolution temperature and time may result in the deterioration of the sericin molecules. At a temperature of 85 °C, more than 10 min were required to reach a sericin solubility of 90%. However, at temperatures of 95 °C and 100 °C, the solubilities of the extracted sericin were similar to that at 90 °C. Therefore, 90 °C was selected as the dissolution temperature for all subsequent examinations.

### 3.2. Rheological Properties of Redissolved Sericins

**Figure 2:**
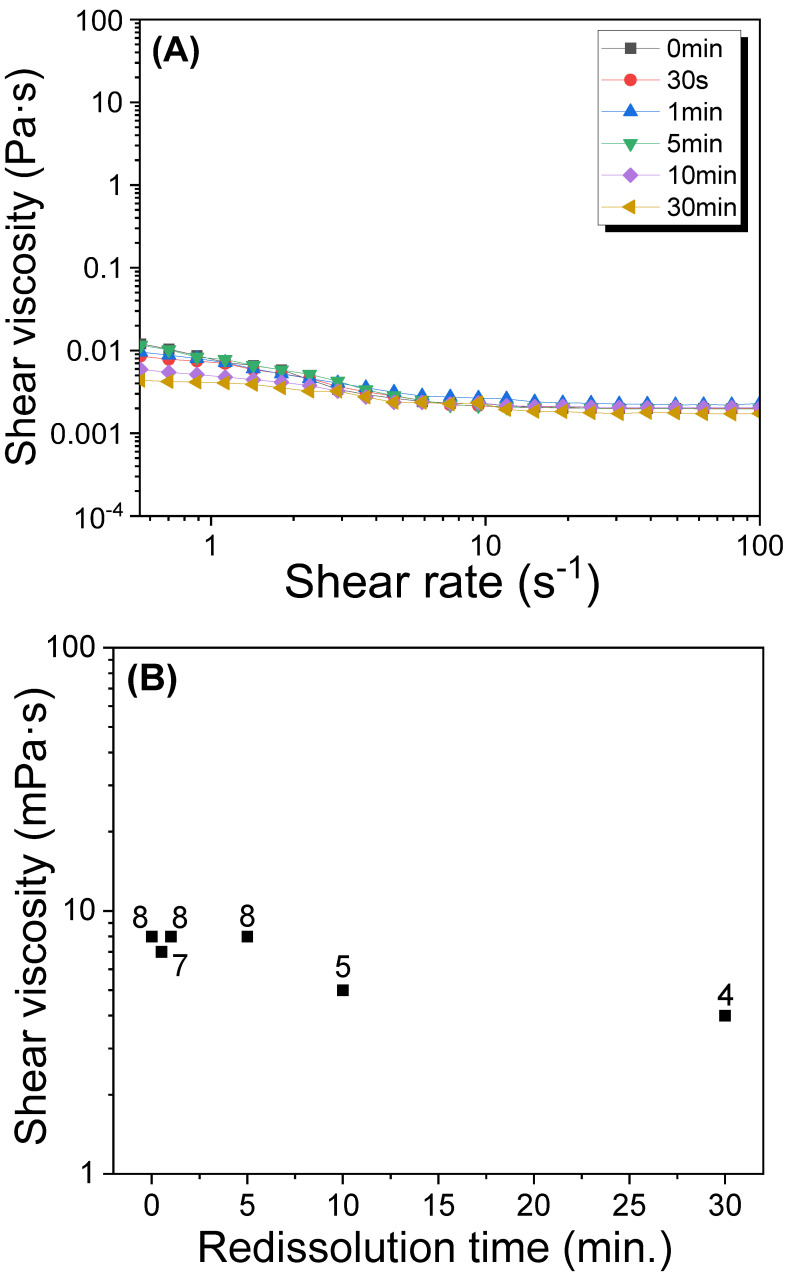
(**A**) Steady-state flow and (**B**) shear viscosity at 1 s^−1^ of 0.3% (*w*/*w*) silk sericin/formic acid solutions prepared by redissolution at 90 °C at different times.

As mentioned previously, the MW of sericin may decrease in water at elevated temperatures. SDS-PAGE [31,32] and liquid chromatography [21,33,34] are two extensively used methods for determining the MW. However, the former method yields excessive broad bands, rendering it a less precise MW measurement method, whereas the latter is an excessively difficult technique in terms of obtaining reliable results. Fortunately, the solution viscosity measurement using a rheometer showed a good relationship with the MW obtained from fast protein liquid chromatography in previous studies [20,21,33]. Therefore, this study performed rheological measurements to evaluate the solution viscosity of redissolved sericin. Figure 2 shows the steady-state flow result (A) and solution viscosity at 1 s^−1^ (B) of the redissolved sericin/formic acid solution for different dissolution times. The extracted (i.e., 0 min) and redissolved sericin/formic acid solutions displayed a slight shear thinning at a low shear rate region (<5 s^−1^). By increasing the dissolution time to 10 min, the shear thinning was less evident. The shear viscosity at 1 s^−1^ of the redissolved sericin/formic acid solution demonstrated this trend more clearly. The viscosity of the sericin/formic acid solutions that were extracted and redissolved was 8 mPa∙s until the dissolution time of 5 min. Moreover, it decreased to 4 mPa∙s when the dissolution time increased to 30 min. This result indicates that molecular degradation of redissolved sericin did not occur until a dissolution time of 5 min.

**Figure 3:**
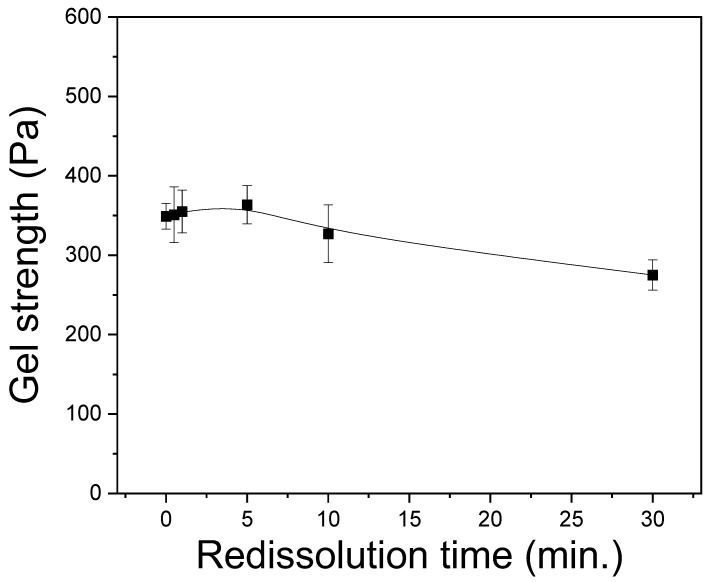
Gel strength of 1.5% (*w*/*w*) silk sericin/formic acid solution prepared by redissolution at 90 °C at different times (n = 5).

The gelation of the sericin solution follows the mechanism of three-dimensional (3D) network structure formation through the intermolecular hydrogen bonding between the sericin molecules [14,25]. As the length of the molecular chain (i.e., MW) of sericin decreases, the three-dimensional network structure becomes weaker, resulting in a decrease in gel strength [21]. Thus, the gel strength of sericin has a good correlation with its MW. Therefore, to reconfirm the MW change in sericin according to the dissolution time, the gel strength of silk sericin at different dissolution times was measured, and the results are exhibited in Figure 3. The sericin gels yielded a gel strength of 349–363 Pa until a dissolution time of 5 min. Thereafter, the gel strength decreased to 275 Pa with an increase in the dissolution time of up to 30 min. This trend is the same as that of the solution viscosity of sericin, as shown in Figure 2. Thus, this result reconfirmed that the MW of extracted sericin experienced minimal change until a dissolution time of 5 min, following which it decreased with a further increase in dissolution time.

### 3.3. Structural Characteristics of Redissolved Sericins

The molecular conformation and crystallinity of silk protein have been extensively studied because they dominate the mechanical properties of silk. The breaking strength of silk fibroin (SF) and sericin films has been found to increase with an increasing crystallinity index [21,25,34]. The post-drawing ability of regenerated SF increases with an increasing crystallinity index [35,36]. Further, the water retention of SF and sericin decreases with an increasing crystallinity index [21,37]. In addition, the crystallinity index of silk proteins increases with an increase in the MWs of fibroin [35] and sericin [21,38].

**Figure 4:**
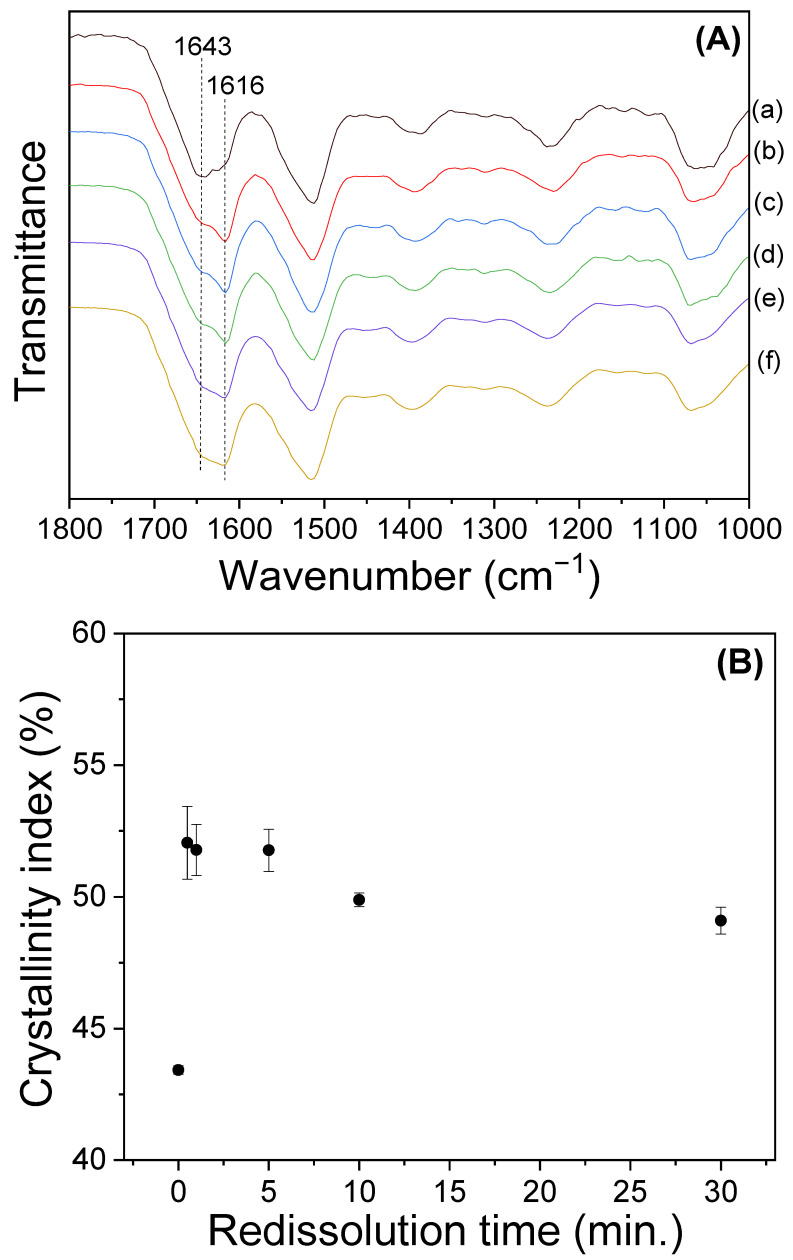
(**A**) ATR-FTIR spectra and (**B**) crystallinity index of silk sericin film prepared by redissolution at 90 °C at different times: (a) 0 min, (b) 30 s, (c) 1 min, (d) 5 min, (e) 10 min, and (f) 30 min (n = 7).

This study examined the molecular conformation and crystallinity index of the redissolved sericin film using FTIR spectroscopy, as can be seen in Figure 4. All sericin films showed an IR absorption peak at 1643 and 1616 cm^−1^, owing to the random coil and β-sheet crystallite, respectively [21,26]. In the case of the extracted sericin film (Figure 4A), the infrared (IR) absorption peak at 1643 cm^−1^ was stronger than that at 1616 cm^−1^, indicating that random coil conformation dominated in this sample rather than β-sheet conformation. However, the redissolved sericin films yielded a stronger peak at 1616 cm^−1^, implying that β-sheet conformation prevailed in these samples. With an increase in the dissolution time to 10 and 30 min, the IR peak at 1616 cm^−1^ became less evident, indicating the decrease in the β-sheet conformation content. The crystallinity index for silk protein [39,40,41] has been extensively utilized to quantitatively investigate the content of molecular conformations in silk proteins. This is because it can be calculated easily and shows a good correlation with the proportion of molecular conformation of silk obtained via deconvolution of the FTIR spectrum [21,42,43].

Figure 4B exhibits the effect of the dissolution time on the crystallinity index of sericin films. The extracted sericin (i.e., 0 min dissolution time) yielded a crystallinity index of 43%. The crystallinity of the sericin film substantially increased to 52% via dissolution and was constant until a dissolution time of 5 min. Thereafter, the crystallinity index decreased to 49% with an increase in the dissolution time to 30 min. 

The increase in the crystallinity of redissolved sericin film compared to the extracted sericin film may be attributed to the crystallization character of sericin; that is, the gelation and crystallization of sericin are accelerated with the decrease in the drying (or storage) temperature [24]. In the case of the extracted sericin, it was extracted at 100 °C and filtered and moved to a drying oven at 60 °C. Consequently, the extraction amount of the sericin aqueous solution was 800 mL and was slowly cooled at room temperature in the filtration system. Thus, this restricted the gelation and crystallization of sericin before drying at 60 °C. Whereas, in the case of the redissolved sericin solution, it was prepared by dissolving at 90 °C, and its amount was 40 mL. Owing to the lower volume and temperature than the extracted sericin solution, the redissolved sericin solution cooled faster at room temperature of the filtration system, resulting in the crystallization of sericin. 

It has been reported that the crystallinity of sericin decreases with the decrease in the MW of sericin [21]. Therefore, the slight decrease in crystallinity at the dissolution times of 10 and 30 min can be due to the decrease in the MW of sericin at these dissolution times. Thus, the lower crystallinity of redissolved sericin at 10 and 30 min reconfirmed the lower MW of redissolved sericin at these dissolution times, as indicated by the results of the solution viscosity and gel strength (Figure 2 and Figure 3).

**Figure 5:**
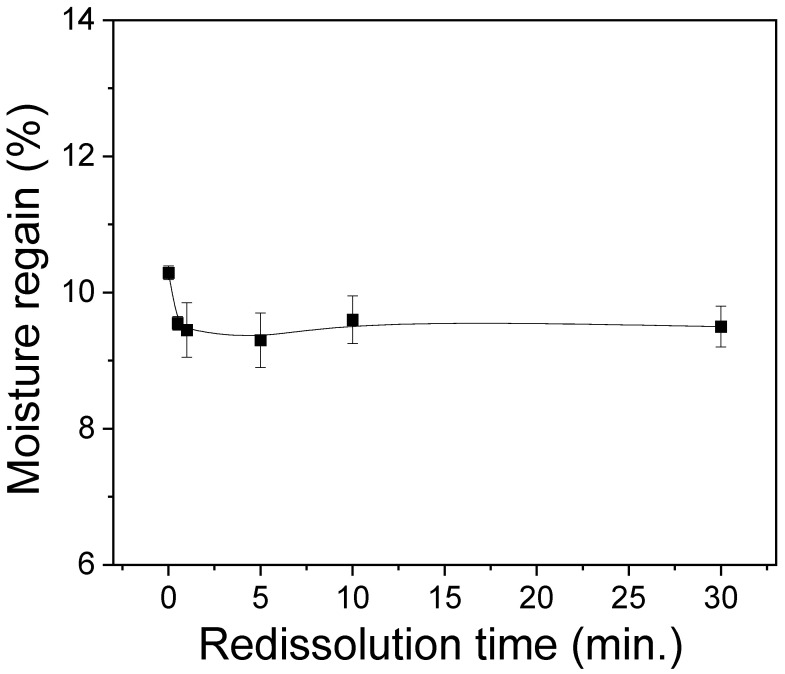
Moisture regains of silk sericin powder prepared by redissolution at 90 °C at different times (n = 3).

Water absorption capacity (or hydrophilicity) are one of important properties of biomedical and cosmetic materials, as it is required for facial mask packs and wound dressing [44]. Therefore, the moisture regain of the silk sericin films was evaluated, and the results are shown in Figure 5. The extracted sericin film (i.e., 0 min) yielded a moisture regain of 10.3%. Following dissolution, the redissolved sericin films showed a moisture regain range of 9–10%. The slight decrease in the moisture regain of the redissolved sericin films compared to the extracted sericin films can be attributed to their higher crystallinities. It has been reported that the moisture regain of silk proteins exhibits a negative correlation with their crystallinity [21,25,37]. 

**Figure 6:**
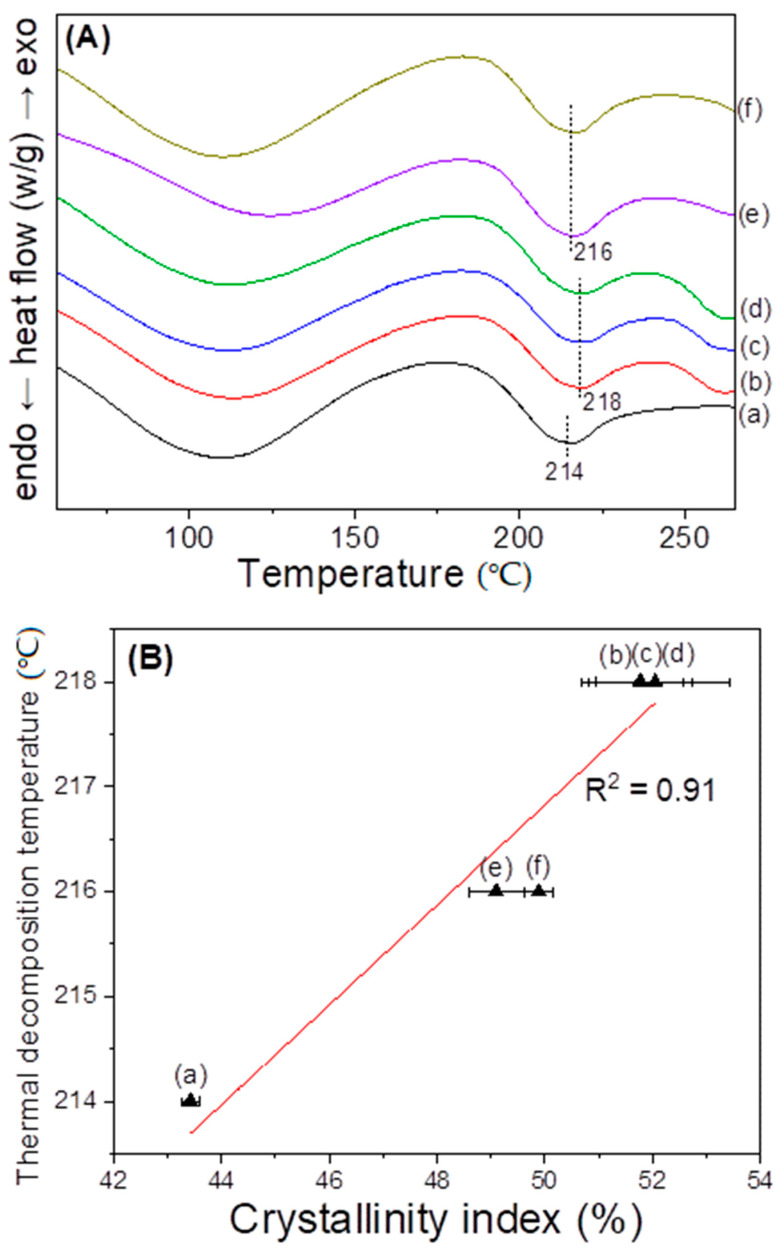
(**A**) DSC thermograms and (**B**) the effect of thermal decomposition temperature on the crystallinity index of silk sericin powder prepared by redissolution at 90 °C at different times: (a) 0 min, (b) 30 s, (c) 1 min, (d) 5 min, (e) 10 min, and (f) 30 min (n = 7).

Figure 6A displays the DSC curve of the sericin film at various dissolution times. All sericin films exhibited a broad endothermic peak at 210–220 °C, owing to the thermal degradation of sericin [42,45,46]. The extracted sericin yielded a thermal degradation peak at 214 °C. Furthermore, the peak shifted to 218 °C at a dissolution time of 30 s and was constant up to the dissolution time of 5 min. Thereafter, the peak shifted to 216 °C. The thermal decomposition was influenced by the MW and crystallinity of silk protein [33,47]. Considering that the MW of the extracted sericin film was the same as that of the redissolved sericin film until 5 min, the peak shift of thermal degradation was due to the difference in crystallinity. Thus, the increase in the crystallinity index of redissolved sericin films resulted in an increase in the thermal decomposition temperature. The slight decrease in the thermal decomposition temperatures at the dissolution times of 10 and 30 min may owe itself to the decrease in the MW and crystallinity of these sericin films compared to other sericin films, as shown in Figure 2, Figure 3 and Figure 4. Figure 6B shows a positive relationship (R^2^ = 0.91) between the crystallinity index and thermal degradation temperature of sericin films, thus reconfirming that crystallinity dominates the thermal degradation temperature of sericin films. 

### 3.4. Cell Viability of Redissolved Sericin

**Figure 7:**
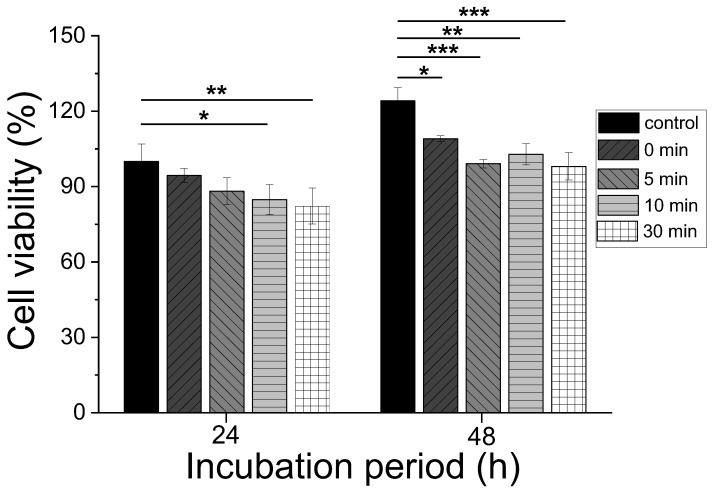
Cell viability of the silk sericin prepared by redissolution at 90 °C at different dissolution times (* *p* < 0.05, ** *p* < 0.01, *** *p* < 0.001/n = 3).

As sericin can be employed for use in biomedical and cosmetic products, including wound dressings, bone substitutes, and mask packs, its cell viability should be examined. The cell viability of the sericin at various dissolution times was examined by performing the CCK test, and the results are shown in Figure 7. For 24 h incubation, with the increase in the dissolution time to 10 min and above, the redissolved sericin films (10 min, * *p* < 0.05; 30 min ** *p* < 0.01) exhibited a significant difference compared with the control. For 48 h incubation, all the sericin films showed a significant difference compared with the control.

**Figure 8:**
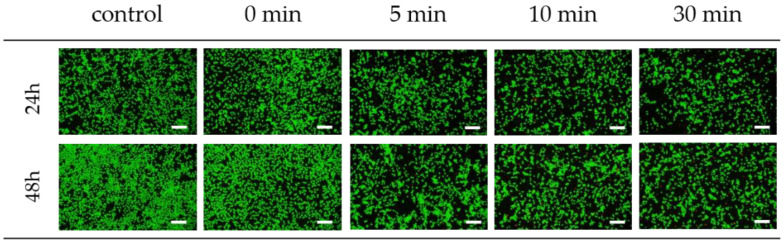
Fluorescence images of the cell viability assay of the silk sericin prepared by redissolution at 90 °C at different times. The white scale bars represent 200 µm.

Fluorescence images of the cell viability assay of the silk sericin in Figure 8 reconfirmed the result of cell viability in Figure 7. All the sericin samples exhibited a considerably greater number of live cells than dead cells, regardless of the dissolution time. This indicated the good cell viability of the sericin samples, although the number of live cells was lower than that of the control at the incubation times of 24 and 48 h. 

According to ISO 10993-5, more than 80% cell viability is considered non-toxic [30,48]. Considering that all sericin films exhibited more than 83% cell viability, the sericin film tested in this study can be concluded to be non-toxic. The non-toxicity of sericin in the present study is consistent with the results in previous reports [49,50,51,52]. Moreover, for both the 24 and 48 h incubation periods, the sericin samples did not show a significant difference between the two. This indicates that the dissolution of the extracted sericin did not affect its cell viability. Furthermore, the MW decrease of sericin that occurred, owing to the dissolution (i.e., 10 and 30 min), did not cause the deterioration of the cell viability of sericin. This result shows good consistency with a previous report that the MW of sericin does not affect the metabolic activity of sericin [21].

Although the sericins are non-toxic, the cell viability of sericins at an incubation time of 48 h showed significant differences (* *p* < 0.05, ** *p* < 0.01, *** *p* < 0.001) compared to the control. Lee et al. reported that the cell viability of silk nonwoven fabric slightly decreased with an increase in sericin content [42]. Kim et al. reconfirmed this result in the differently prepared silk nonwoven fabrics [53]. These reports indicate that the cell viability of sericin is not excellent compared to silk fibroin. Although the exact reason for the lower cell viability of sericin than the control cannot be elucidated in the study, the hydrophilicity of sericin might be one of the reasons for that. That is, because the sericin is hydrophilic, it might be very partially extracted in the culture medium, resulting in a slightly negative effect on cell viability. 

## 4. Conclusions

This study extracted sericin from silkworm cocoons, which were then dissolved in hot water to prepare redissolved sericin. The structure and properties of redissolved sericin were investigated comparatively with those of the extracted sericin. The solubility of the extracted sericin at 90 °C for 5 min reached 95.1%. With further increases in the dissolution temperature (i.e., 95 °C and 100 °C), the solubility did not change considerably. Furthermore, the solution viscosity, gel strength, crystallinity, and thermal decomposition temperature of redissolved sericin were observed as remaining constant until a dissolution time of 5 min, indicating that its MW did not change until 5 min. Moreover, the dissolution time did not influence the cytocompatibility of redissolved sericin. 

These results indicate that the extracted sericin can be stored in a dry state and used by dissolving it in hot water (e.g., 90 °C for 5 min) to prepare the sericin aqueous solution. This implies that sericin can be used in biorelated applications without the problem of storage. Therefore, it is believed that the findings presented in this study will accelerate the use of sericin in biorelated applications.

## Data Availability

The data presented in this study are available on request from the corresponding author.
